# Chairside CAD/CAM Materials: Current Trends of Clinical Uses

**DOI:** 10.3390/biology10111170

**Published:** 2021-11-12

**Authors:** Giulio Marchesi, Alvise Camurri Piloni, Vanessa Nicolin, Gianluca Turco, Roberto Di Lenarda

**Affiliations:** Department of Medical, Surgical, and Health Sciences, University of Trieste, 34127 Trieste, Italy; alvise.camurripiloni@gmail.com (A.C.P.); nicolin@units.it (V.N.); gturco@units.it (G.T.); rdilenarda@units.it (R.D.L.)

**Keywords:** CAD/CAM, chairside, resin, ceramic, block, hybrid ceramic

## Abstract

**Simple Summary:**

The interest in chairside Computer-aided design/computer-assisted manufacturing (CAD/CAM) restorations has increased the diversity of the restorative material. The promise of accurate, esthetic restorations delivered rapidly to the patient has manly benefits for clinicians in the light of minimally invasive dentistry dictates. New materials have been developed by the industry in order to offer ceramic, composite, and hybrid materials with optimized properties, suitable mechanical and aesthetic features. However, this comes at the expense of making the application more complicated. This article is aimed at providing an overview regarding the main advantages and disadvantages of the CAD/CAM chairside materials.

**Abstract:**

Restorative materials are experiencing an extensive upgrade thanks to the use of chairside Computer-aided design/computer-assisted manufacturing (CAD/CAM) restorations. Therefore, due to the variety offered in the market, choosing the best material could be puzzling for the practitioner. The clinical outcome of the restoration is influenced mainly by the material and its handling than by the fabrication process (i.e., CAD/CAM). Information on the restorative materials performances can be difficult to gather and compare. The aim of this article is to provide an overview of chairside CAD/CAM materials, their classification, and clinically relevant aspects that enable the reader to select the most appropriate material for predictable success.

## 1. Introduction

In dentistry, the synergic effect of the introduction of digital technologies on one side, and the evolution of materials with suitable mechanical and aesthetic features on the other side, has led to a profound change in prosthetic restorative dentistry.

More and more colleagues are using Computer-aided design/computer-assisted manufacturing (CAD/CAM) chairside technology, and manufacturers are increasingly expanding their material offering with the aim of reducing armchair operating times while having high standards of precision and aesthetics.

CAD/CAM technology is a digital workflow that requires three steps:

1. Impression with software associated with an over-the-counter scanner or intraoral camera;
2. Digital data processing using a program to delimit dental preparation, occlusion, and restoration contacts;
3. Restoration production is designed using subtractive manufacturing processes, which require milling the desired restoration starting from a block of material.

The first generation of CAD/CAM machinery appeared in the market in the 1980s, and it was only able to design and produce ceramic indents. Nowadays, the main chairside systems (e.g., PlanScan, Carestream, or CEREC) are using a “full-digital workflow”, which can produce different prosthetic devices such as inlays/onlays, veneers, endo-crowns, bridge crowns, and implant abutments, to name some [1,2].

For the first 15 years or so, the feldspathic ceramics (Vita Mark I, Vita Zahnfabrick, Bad Sackingen, Germany) was the only one available for CEREC. Feldspathic ceramics were modified in order to be used for small occlusal inlays. Then, the need for superior mechanical properties induced the development of new materials that could extend the indications of CAD/CAM restorations (onlays, crown). Examples of these microstructures are leucite, lithium disilicate glass-ceramics, and zirconia. Some of the aforementioned materials could be found in a pre-crystallized stage in order to guarantee rapid milling. Nevertheless, a post-milling crystallization is mandatory to access the final shade and the proper mechanical strength. In chairside technology, the use of monolithic materials is preferred. Indeed, digital anatomical modeling allows having minimal thicknesses avoiding the main mechanical complication of “bi-layer” systems (i.e., the chipping of coating ceramics) [3].

Recently CAD/CAM resin composite and hybrid ceramic materials have been introduced. Benefits are easy intraoral repair with light-cured restoratives and a faster production rate since firing is not needed [4].

CAD/CAM materials for chairside production can be classified according to the composition of the materials (Table 1). Each category offers mechanical and physical features and unique indications for specific clinical applications. The CAD/CAM chairside system represents only the production process, while the clinical outcome of the restoration is mainly influenced by the type of material chosen to carry out the restoration and the way in which it is managed. (Table 2). The purpose of this article is to offer a quick, summary overview of these constantly evolving materials, which today propose diverse restoration methods to the clinician.

## 2. Materials and Methods

### 2.1. Adhesive Ceramics

Glass-ceramics materials were the first to be developed for CAD/CAM systems. They are characterized by a significant amount of glass components, which makes them among the most translucent and aesthetic materials, providing a “chameleon” effect that allows the restoration to mimic the color of the existing tooth. The first material used with chairside was Vita Mark I feldspathic pottery in 1985, and since today, this type of class of ceramic materials has had a remarkable increase in bending force resistance from 125 to 375 MPa [5,6].

### 2.2. Feldspathic and Leucite-Reinforced Ceramics

Today two types of ceramics of this category are basically available on the market. One group consists of feldspathic ceramics (Vitablocs Mark II, Vita Zahnfabrik; Bad Sackingen, Germany, and CEREC blocks, (Dentsply Sirona; Bensheim, Germany); the other group is composed of leucite-reinforced ceramic (IPS Empress CAD, Ivoclar Vivadent; Schaan, Liechtenstein; LRF block, GC). Feldspathic and leucite-reinforced ceramics present a prevalence of the glass phase (55% to 70%), which makes them among the most translucent and most aesthetic ceramic materials [7].

Currently, it is indicated with regard to these materials to use them for partial adhesive restorations such as veneer and onlay, overlay. For traditional restorations such as bridges, it is indicated up to 3 elements in the front area and individually up to the level of the second premolar [8].

These materials are available as monochrome blocks or as polychromatic blocks (Vitablocs Triluxe forte and Vitablocs RealLife, Vita ZahnFabrik) and possess translucency and a chromatic range that simulates one of the native dental tissues: dentine and enamel [9]. Since adhesive ceramic materials have been available for more than 25 years, the results and observations of several long-term clinical trials are nowadays available. The average survival rate for inlay and onlay leucite restorations in the posterior area is 96.7% after 9 years [10].

A recent meta-analysis estimated survival rate between 92% and 95% for 5811 adhesive restorations (onlays, overlays) with feldspar ceramics at 5 years and 91% at 10 years for 2154 restorations [11]. No significant difference in the survival rates has been reported based on the number of dental surfaces restored [12].

### 2.3. Lithium Disilicates and Zirconia-Reinforced Lithium Silicates

Glass ceramics with improved resistance properties represented a paramount development in the field of fixed prostheses. IPS e.max CAD (Ivoclar Vivadent) was introduced in 2006 as a lithium disilicate with a bending resistance over 350 MPa and significantly greater fracture resistance than previous adhesive glass ceramics [13]. It is characterized by a glass matrix embedding small needle-shaped crystals with dimensions around 0.2–1 µm. In the CAD/CAM method, lithium disilicate is supplied only in partially crystallized form (purple color), in which the block consists of lithium metasilicate (Li2SiO3) and for the rest by the crystallized nucleus of lithium disilicate (Li2Si2O5) that provide a “soft” state (bending resistance of 140 MPa). This makes the block easier to mill while reducing the wear of the milling cutters [13]. Post milling, the material must be subjected to a two-stage firing cycle in a ceramic furnace for 10 min and involves the complete crystallization and the transformation of the metasilicate into lithium disilicate with an increase in bending resistance over 440 MPa [14]. The crystallization kinetics and microstructure of the disilicate are significantly influenced by thermal and mechanical processes. According to some studies, heating the material for longer times (14 min at 840 °C) leads to improving its mechanical features, increasing its hardness its elastic modulus [15]. Lithium disilicate is indicated for partial adhesive restorations such as veneer and onlay, overlay, and for crown restoration. Several studies have shown that the performance of monolithic crowns in lithium disilicate after 8 years has shown survival rates of up to 94.8% and 10 years at 96.7%; reducing significantly the incidence of complications due to chipping [16,17,18,19]. A recent clinical study based on monolithic lithium disilicate crowns applied on the chairside showed a survival rate of 83.5% and a complication-free rate of 71.0% after 10 years [20]. As for the use of bridges, they can be used up to 3 elements in the posterior sector, and according to a clinical trial at 10 years, there would be no difference in terms of survival between this material and ceramic-metal ones [21].

Lithium disilicate has also demonstrated the possibility of being used as a single crown for implant restorations and as a single hybrid implant abutment supported by titanium tibase [22,23].

Another machinable dental glass-ceramic block composed of lithium disilicate is the Amber® Mill (HASS, Gangwon-do, Korea), which exhibits a flexural strength of around 300 MPa. It is indicated for table tops, veneers, inlays/onlays, and crowns or can be used for anterior and premolar 3-unit bridges.

This material also allows being used adhesively for veneer, overlay, and onlay. In the posterior sectors, a minimum thickness of 1.5 mm is generally recommended to have a proper distribution of stresses [24]. The current tendency in tooth wear treatment is to avoid any tooth tissue preparation (“No prep”) using very low thickness prostheses (table tops) with minimum thicknesses that range from 0.5 to 1 mm [25,26].

A new generation of high-strength CAD/CAM ceramics introduced since 2012 is represented by zirconia reformed lithium silicate (ZLS) in which the glass matrix is reinforced with lithium silicate crystals, 4–8 times smaller than those of lithium disilicate, to which a tetragonal zirconia component (10% by weight) is added in order to improve its mechanical properties [27].

At present, this type of glass ceramic is supplied in two different ways from manufacturers in presintered blocks (Vita Suprinity PC, Vita Zahn Fabrick) or in already sintered blocks (Celtra Duo, Dentsply Sirona). Such ceramic glass materials are suitable for the restoration of individual adhesives (veneer, onlay, overlay) in the anterior and posterior zone. According to some authors, these materials would be more resistant to fracture, easier to be milled (especially the presintered systems), and easier to be polished to the armchair [28,29]. The sintered block (Celtra Duo) comes in an already fully crystallized state that can be polished by hand or have a further heat treatment before delivery. The hand polishing of the restoration improves the bending resistance up to 210 MPa, while the heat treatment allows obtaining a further improvement up to 370 MPa. Currently, only a few clinical works are available in the literature, but the data show that Celtra Duo in adhesive restorations in the anterior teeth, premolar, and molar (92 restorations in 71 patients) exhibit a 3 years survival rate of 99% [30].

In 2021 Dentsply Sirona is introducing a new advanced lithium disilicate CAD/CAM blocks (Tessera, Dentsply Sirona). This ceramic is characterized primarily by the fact that it can be fired exceptionally quickly. The glaze firing takes only four and a half minutes, and the fast firing time is made possible by the new composition of the ceramic composed of disilicate of lithium and virgilite, a lithium aluminum silicate. It is indicated in the anterior and posterior region for crown, inlay, onlay, and veneer.

### 2.4. Resin Composite Materials

Although introduced in the 2000 s, CAD/CAM resin composite materials have become very popular in chairside. The Paradigm MZ100 (3M Oral Care, Seefeld, Germany) was the first resin composite introduced in the market in CAD/CAM blocks with the possibility of different dyes and with a percentage of filler (silica zirconia 0.6 µm in diameter) up to 85% by weight. The bending strength reported for the Paradigm MZ100 is 157 MPa, similar to the bending resistance of feldspathic ceramic materials [31].

According to some authors, this material, if adhesively cemented, would have a proper mechanical resistance even at reduced thicknesses counterpoised by a limited resistance to abrasion [32,33].

Starting from 2016, the Brilliant Crios (Coltene/Whaledent; Coltène, Alstatten, Switzerland) this material is a reinforced composite containing amorphous silica particles (<20 nm) and glassy ceramic barium particles (<1.0 µm) embedded in a cross-linked methacrylate matrix. The manufacturer reports a filling of 70.7% by weight and a filling of 51.5% by volume, which translates with a bending resistance of 198 MPa and an elastic modulus of 10.3 GPa. The elastic modulus, similar to that of dentine, is suggested to minimize the concentration of stress in the restoration and avoid fractures. The indications for these materials are those for onlays, overlays. In the literature, there is minimal clinical research on CAD/CAM composites, the average survival rate at 10 years is 95% [34].

Tetric CAD (Ivoclar Vivadent, Liechtenstein) is an aesthetic composite with resin matrix composed of Bis-GMA, Bis-EMA, TEGDMA, UDMA nano-filled 70% with barium glass and silicon dioxide. According to the data provided by the production company, the bending resistance is 273.8 MPa, an elastic modulus of 10.2 GPa.

LuxaCam Composite (DMG, Hamburg, Germany) consists of a composite matrix of special polymers in which silicate glass filling substances are embedded. The ratio between the two materials is optimized so that the mechanical values (164 MPa flexural strength and 10.1 GPa elasticity module) are as close as possible to the ones exhibited by the natural tooth. Following manufacturer’s indications, these materials are suitable for table tops, inlays, onlays, veneers, partial crowns, crowns, and bridges (also multi-unit, up to three bridge units).

Grandio Blocks (VOCO GmbH, Germany) is a highly filled (86%) CAD/CAM restorative material that is based on a nanoceramic hybrid technology. It has a flexural strength of 250–290 MPa and 15.5 GPa elastic modulus with a coefficient of thermal expansion similar to the dentin and enamel.

### 2.5. Hybrid Ceramics

Hybrid ceramics are a new category of CAD/CAM chairside materials that have been designed to take advantage of the reduced fragility and increased fracture resistance typical of composite resins combined with the unique aesthetic characteristics of the ceramic materials. At present, they are divided into two different classes: nanoceramic resin blocks that are made industrially by high-temperature and high-pressure processes, in which the composite resin (generally present Bis-GMA, UDMA, UTMA, and Bis-EMA) is coupled with a ceramic filler (up to 80% by weight) and polymer-infiltrated-ceramic network (PICN) blocks in which the ceramic structure at (86% by weight) is industrially infiltrated by composite resin (14% by weight) [35].

Hybrid ceramics have proved to be easier to be milled and without the need for additional thermal cycles. Moreover, they exhibit high bending resistance and the possibility of being used even at reduced thicknesses [36]. With regard to nanoceramic materials, the first to be introduced on the market is Lava Ultimate (3M Ultimate, 3M Oral Care), which contains particles of silica (20 nm) and zirconia (4 to 11 nm) up to 80% in weight. According to the manufacturer, an advantage for nanoceramic material over CAD/CAM composite blocks is the ability to maintain a high shine surface finish. The manufacturer reports a bending force resistance of about 200 MPa [37]. Nanoceramic blocks are used for occlusal coverage or inlays/onlays in the posterior zone, by the way, not for the realization of crowns [38]. At the moment, we only have a 5-year clinical study showing that this adhesively cemented CAD/CAM nanoceramic material for overlays and onlays has similar behavior to that of feldspathic ceramics in the posterior sector [39].

Another nanoceramic on the market is Cerasmart (GC America, Alsip IL, USA), which is a block characterized by an organic resin matrix containing particles of silica filler and barium glass up to 71% by weight. The resistance to bending force is reported for Cerasmart of 230 MPa, and this material is indicated for the restoration of a single tooth (cuspidal coatings, indents, and crowns). Recent clinical work demonstrates its use with suitable results for two-year inlays and onlays compared to lithium disilicate [40].

On the market, there are numerous other materials belonging to this category, including Block HC (Shofu, Kyoto, Japan) characterized by a resinous matrix based on UDMA and TEGDMA filled 68% with silica and zirconia silicates. The resistance to bending force reported by the manufacturer is 170–190 MPa and an elastic modulus of 7.8 GPa.

The Katana Avencia Block (Kuraray, Noritake, Dental) is a hybrid ceramic consisting of UDMA, and methacrylate polymers filled 62% with silica oxide and aluminum with bending force resistance of 230 MPa and compression force resistance of about 680 MPa following manufacturer data.

From experience obtained with In-Ceram system infiltrated glass ceramics, in 2013 Vita Zahnfabrik introduced the Enamic, which can be considered a real new type of hybrid ceramic. This type of material is called PICN (polymer-infiltrated-ceramic network), in which the ceramic structure reinforced with leucite and zirconia (86% by weight) is infiltrated by a resin base (UDMA, TEGDMA) (14% by weight).

The mechanical properties of the material are at an intermediate level between that of adhesive ceramics and that of highly filled composites. It has an elastic modulus of around 30 GPa [6]. The ceramic component improves wear resistance; however, it can make the material more fragile and susceptible to fracture. The polymer mesh improves the fracture resistance of the material thanks to its ability to undergo plastic deformation. This material is suitable for inlays, onlays, and crowns, and the manufacturer claims a bending resistance of about 130 MPa [41]. The manual final polishing of this material is fast and allows to obtain surfaces with a polishing comparable to the ceramic materials one. The final modification is limited to the use of supercolors and cold glazing. This type of material must be cemented in an adhesive way to the tooth structure for both overlays and crowns.

Clinical trials on these materials are unfortunately limited due to their recent introduction on the market; at the moment, only a few 3-year clinical works are showing a survival rate of 97.4% for inlays and 95.6% for overlays [42].

Mazic Duro (Vericom, co., Gangwon-do, Korea) is a radiopaque hybrid ceramic intended for use in dental CAD/CAM milling procedures. The polymeric matrix consists of a high degree of cross-linking monomer. The inorganic fillers are barium, aluminiumsilicate, silicon dioxide, and zirconia. The total content of inorganic fillers is approximately 80%, and particle size is between 0.01 and 1 mm.

### 2.6. Zirconia

Zirconia is a heterogeneous polycrystalline ceramic and is characterized by excellent mechanical properties (flexural strength 500–1200 MPa, elastic modulus of 210 GPa) and suitable aesthetic properties but is not susceptible to traditional acid bite procedures [43,44]. Both in vivo and in vitro, it has excellent biocompatibility, reduced plaque retention compared to titanium, and has the lowest rate of wear against the antagonist among the various integral ceramics [45].

From a chemical-physical point of view, zirconia is a metal oxide characterized by polymorphism and allotropy and is present in nature in 3 different crystalline structures: cubic (from the melting point to 2680 at 2370 °C), tetragonal (2370 to 1170 °C), and monocline (1170 °C at room temperature).

Zirconia is mainly used in both tetragonal and cubic phases. At room temperature after post-sinter cooling, zirconia crystals may experience irreversible spontaneous transformation to the monocline form with an increase in crystal volume of 4–5% and the generation of high compressive stress within the material that is called phase transformation toughening (PTT). PTT is exploited in prosthetics since zirconia has the possibility to block or at least slow down the progression of micro-cracks and fractures within the material, transforming from the tetragonal to the monocline phase [46]. At the industrial level, zirconia is stabilized with oxides such as yttrium, magnesium, cerium, and lanthanum, and these oxides can be lost as a result of trauma, superficial modifications (occlusal adjustments, finishing), and aging of the material. Zirconia is an opaque restoration material with aesthetic and less captivating properties than glass ceramics, suitable for masking dyschromic stumps or stumps with metal restorations. Recently, 30–35% cubic zirconia with high translucency has been introduced. Beyond the improved optical properties, however, it has significantly lower mechanical resistance values with residence to the bending between 500 and 900 MPa due to the size of cubic granules that are larger than those tetragonal [47,48].

The zirconia chairside provides for the processing only soft-machining or the use of presintered zirconia that makes it more easily breakable, reducing processing times, the wear of the machinery creating negligible internal porosity [49]. On the other hand, this procedure provides for zirconia to be milled with an oversizing of 25% of the final volume, which will then have to be compensated during the sintering phase. This material is indicated for the manufacture of single crowns, implant abutment, and it is possible with chairside blocks to create bridges up to three elements. The minimum thickness of monolithic restorations is 0.5 mm [50].

The first block introduced on the market in 2016 was Cerec zirconia (Dentsply Sirona), characterized by multilayer pre-colored zirconia blocks. In the same period, an induction furnace for sintering (SpeedFire, Dentsply Sirona; Bensheim, Germany) was introduced, which significantly reduced the sintering time to less than 20 min, thus allowing the design, processing, and delivery of the restoration in a single appointment. Bending resistance and fracture resistance is at least three times higher than that of ceramic adhesive glass materials. The resistance to the fracture force reported for zirconia is more than 1000 MPa. The high strength of zirconia allows both traditional cementation and adhesive cementation, although there is no evidence in the literature of which is the cementation method that guarantees the greater resistance to retention. Being a new category of CAD/CAM chairside materials, formulations with greater translucency and sufficient strength to be cemented rather than glued with adhesive techniques are being developed. Newer materials for CAD/CAM application such as Katana Zirconia (Kuraray Noritake Dental; Tokyo, Japan), Mazic Zir HT, Mazic Zir Ultra HT (Varicom co., Korea), LuxaCam Zircon HT Plus (DMG, Hamburg, Germany), IPS e.max and zirCAD (Ivoclar Vivadent), were introduced with better translucency and aesthetic result. However, to date, there is limited clinical evidence regarding these materials.

### 2.7. Resins

Interim prostheses represent a paramount part of the fixed prosthodontic treatment and are usually made from conventional resin materials such as poly(methyl), poly(ethylene), and bis-acrylate composite resins. Polymethyl methacrylate (PMMA)-based polymers are pre-polymerized without incorporation of fillers and stored until usage. Their mechanical properties depend mainly on their chemical composition and their cross-linked structure. Structural properties may be better than the ones of the conventional resins, such as low mechanical stability due to porosity, absence of voids, and lower polymerization shrinkage that occur during mixing, packing, and setting. Interim prostheses can be made in a shorter chairside time. These PMMA-based polymers could also be used for long-term interim prostheses. The most common are:

LuxaCam PMMA DMG, Hamburg, Germany) is a copolymer based on PMMA >99%.

ArtBlock Temp (MERZ Dental GmbH, Germany) is composed of highly cross-linked interpenetrated OMP-N (Organic Modified Polymer Network) without inorganic fillers, has a flexural strength of more than 90 MPa and an elastic modulus of 2680 MPa.

TelioCAD (Ivoclar Vivadent, Schaan, Liechtenstein) is composed of PMMA and is used to produce full-contour single-tooth and multiple-unit temporary restorations using CAD/CAM technology. It has a flexural strength of 130 MPa and a flexural modulus of 3200 MPa, a Vickers hardness of 190 HV, and a water absorption less than 28 μg/mm^3^.

CAD Temp (Vita Zahnfabrik, Germany) is a particular fiber-free, homogeneous, high-molecular, and cross-linked acrylate polymer with a microscopic filler named MRP (microfiller reinforced polyacrylic). In this material, developed by VITA, inorganic microfillers are polymerized into the network and a completely homogeneous, methyl methacrylate-free material, which exhibits superior material quality and outstanding abrasion resistance. It has a flexural strength higher than 80 MPa and an elastic modulus of 2800 MPa.

Mazic Pro (Vericom co., Korea) is a provisional hybrid composite for CAD/CAM restoration containing 10 nm ceramic filler and pearl-type polymer with flexural strength 170 MPa and surface hardness equal to 25 HV.

## 3. Conclusions

Due to the various materials available nowadays, the term chairside CAD/CAM restoration is not fully explicative of the actual restorations. Understanding the material features, their pros and cons, and how they have to be handled will play a paramount role in choosing the specific material for a particular clinical situation.

The outcome of the clinical treatment is strictly related to the attention paid to choosing the unique properties and features of the various categories of CAD/CAM materials. The success of chairside restorations depends on several factors such as material selection, restoration design, occlusion, and cementation.

At the moment the chairside CAD/CAM materials are recent and there are only limited evidence and narrow clinical experience of such material use. Some material offered few years ago have been withdrawn of the market. The clinician must choose the material best suited to his works habits.

## Figures and Tables

**Table 1:** Summary of the products presented in the study.

Categories	Description	Commercial Name	Manufacturer
Adhesive ceramic	Feldspathic ceramic	Vitablocs Mark II CEREC Blocs Vitablocs Triluxe Vitablocs RealLife	Vita Zahnfabrik Dentsply Sirona Vita Zahnfabrik Vita Zahnfabrik
	Leucite-reinforced ceramic	IPS Empress CAD Initial LRF Block	Ivoclar Vivadent GC
	Lithium disilicate	IPS e.max CAD Amber Mill Tessera	Ivoclar Vivadent HASS Dentsply Sirona
	Lithium silicate zirconia reinforced	Celtra Duo	Dentsply Sirona
Composite resin	Bis-GMA composite	Paradigm MZ100 Brilliant Crios Grandio Blocks LuxaCam composite Tetric CAD	3M Coltene/Whaledent Voco DMG Fabrik Ivoclar Vivadent
Hybrid ceramic	Nanoceramic	Lava Ultimate Cerasmart Shofu Block HC Cerasmart Mazic Duro Avencia Block	3M GC Shofu GC America Vericom co. Kuraray Noritake Dental
	PICN	Enamic	Vita Zahnfabrik
Zirconia	Tetragonal zirconia	CEREC Zirconia e.max ZirCAD Katana Zirconia Block Mazic Zir LuxaCam Zircon HT Plus	Dentsply Sirona Ivoclar Vivadent Kuraray Noritake Dental Vericom co. DMG Fabrik
Resin	PMMA	TelioCAD Cad Temp Mazic Pro LuxaCam PMMA ArtBlock Temp	Ivoclar Vivadent Vita Zahnfabrick Vericom co. DMG Fabrik MERZ

Coltene Whaledent (Alstatten, Switzerland); Dentsply Sirona (Bensheim, Germany); DMG Fabrik (Hamburg, Germany); GC (Tokyo, Japan); GC America (Alsip IL, USA); HASS (Gangwon-do, Korea); Ivoclar Vivadent (Schaan, Liechtenstein); Kuraray Noritake Dental (Tokyo, Japan); MERZ Lütjenburg, Germany); Shofu (Kyoto, Japan);Vericom co. (Gangwon-do, Korea); Vita Zahnfabrick (Bad Sackingen, Germany); Voco (Cuxhaven, Germany); (3M Oral Care (St. Paul, Mn, USA).

**Table 2:** Features and indications for the use of the chairside CAD/CAM materials.

Material/Characteristics	Feldspathic and Leucite-Reinforced Ceramic	Lithium Disilicate and Zirconia-Reinforced Lithium Silicates	Resin Composite	Hybrid Ceramic	Zirconia	Resin
Microstructure	Glassy matrix + Crystalline loads	Glassy matrix + Crystalline loads	Inorganic fillers in resin matrix	Ceramic nanoparticles in resin matrix Ceramic Network infiltrate of polymer	Polycrystalline	Resin polymers
Optic properties	Excellent	Good	Medium	Good	Good	Weak
Bonding aptitude	Excellent	Excellent	Excellent	Excellent	Medium/weak	Excellent
Advantages	Clinical experience, Esthetics, Wide range shade, Translucidity	Clinical experience (e.max), Esthetic, Mechanical strength, Wide range shade, Translucidity	Rapid milling, Direct composite reparation, Mechanical properties	Rapid milling, Direct composite reparation, Mechanical properties	Mechanical strength	Rapid milling, Direct composite reparation, Mechanical properties
Disadvantages	Relative fragility	Less than conventional ceramic	Optical properties	Optical properties	Translucidity	Esthetic
Indications for the use	Veneer, inlay, onlay, overlay, crown, bridge anterior	Veneer, inlay, onlay, overlay, crown, bridge anterior and posterior, abutment	Veneer, inlay, onlay, overlay, crown, bridge of small extent	Veneer, inlay, onlay, overlay, crown (except Lava Ultimate)	Crown, bridge, abutment	Temporary restorations: Veneer, inlay, onlay, overlay, crown, bridge of small extent

## Data Availability

The study did not report any data.
